# A manipulative thermal challenge protocol for adult salmonids in remote field settings

**DOI:** 10.1093/conphys/coaa074

**Published:** 2020-09-14

**Authors:** Daniel S Donnelly, Vanessa R von Biela, Stephen D McCormick, Sarah M Laske, Michael P Carey, Shannon Waters, Lizabeth Bowen, Randy J Brown, Sean Larson, Christian E Zimmerman

**Affiliations:** U.S. Geological Survey, Alaska Science Center, Anchorage, AK 99508, USA; U.S. Geological Survey, Alaska Science Center, Anchorage, AK 99508, USA; U.S. Geological Survey, Leetown Science Center, Conte Anadromous Fish Research Laboratory, Turner Falls, MA 01376, USA; Department of Biology, University of Massachusetts, Amherst, MA 01003, USA; U.S. Geological Survey, Alaska Science Center, Anchorage, AK 99508, USA; U.S. Geological Survey, Alaska Science Center, Anchorage, AK 99508, USA; U.S. Geological Survey, Western Ecological Research Center, University of California, Davis, CA 95616, USA; U.S. Geological Survey, Western Ecological Research Center, University of California, Davis, CA 95616, USA; U.S. Fish and Wildlife Service, Fairbanks, AK 99701, USA; Alaska Department of Fish and Game, Anchorage, AK 99518, USA; U.S. Geological Survey, Alaska Science Center, Anchorage, AK 99508, USA

**Keywords:** Captive holding, Chinook salmon, heat stress, portable, temperature control

## Abstract

Manipulative experiments provide stronger evidence for identifying cause-and-effect relationships than correlative studies, but protocols for implementing temperature manipulations are lacking for large species in remote settings. We developed an experimental protocol for holding adult Chinook salmon (*Oncorhynchus tshawytscha*) and exposing them to elevated temperature treatments. The goal of the experimental protocol was to validate heat stress biomarkers by increasing river water temperature from ambient (~14°C) to a treatment temperature of 18°C or 21°C and then maintain the treatment temperature over 4 hours within a range of ±1.0°C. Our protocol resulted in a mean rate of temperature rise of 3.71°C h-1 (SD = 1.31) to treatment temperatures and mean holding temperatures of 18.0°C (SD = 0.2) and 21.0°C (SD = 0.2) in the low- and high-heat treatments, respectively. Our work demonstrated that manipulative experiments with large, mobile study species can be successfully developed in remote locations to examine thermal stress.

## Teaser

A portable experimental system was developed for remote field settings, capable of holding large fish for several hours, while controlling for precise water temperatures. This system was used to conduct a thermal challenge experiment using adult Chinook salmon along the Yukon River in Alaska, USA.

## Introduction

Increasing water temperature is a widespread global concern for fisheries managers (Wrona *et al.*, 2006; Mantua *et al.*, 2010). Scientists and managers must often rely on correlative studies to understand and anticipate changes in fish populations from changing thermal regimes of rivers and lakes. Manipulative experiments provide stronger evidence for identifying cause-and-effect relationships than correlative studies, but protocols for implementing experiments in remote field settings are lacking. Arctic and subarctic ecosystems especially require protocols that permit experimental manipulation of water temperature in remote locations given the disproportionate effects of a changing climate (Post *et al.*, 2019) coupled with remote landscapes and large fish species.

Declining salmon returns in Alaska over the past several decades have highlighted a need to understand factors that may be suppressing population abundances (ADF&G, 2013; AYK-SSI, 2013; USFWS, 2016). Temperature can be a major driver of salmon population dynamics by influencing the success of spawning migrations. For example, when water temperature approaches upper thermal limits pre-spawning mortality of migrating adult salmon can be high (Keefer *et al.*, 2008; Mathes *et al.*, 2010), exceeding 90% in some cases (Hinch *et al.*, 2012). Temperatures in the mainstem Yukon River and its tributaries in Alaska over the past decade have frequently reached or exceeded temperatures associated with stress and mortality in salmonids (18–21°C) (Richter and Kolmes, 2005; Strange, 2010; Zuray, 2010; Carlson and Edwards, 2017). As a result, fishery managers have voiced concern regarding heat stress related mortality in adult Chinook salmon (*Oncorhynchus tshawytscha*) returning to the Yukon River. Research is needed to understand the impact of increasingly elevated water temperatures on Chinook salmon.

In response to concerns over thermal stress contributing to premature mortality in returning adult Yukon River Chinook salmon, a large-scale study was conducted to look for physiological indicators of thermal stress, using heat stress biomarkers (gene transcription, heat shock protein 70 abundance) measured in non-lethal muscle biopsies taken from salmon throughout the drainage (von Biela *et al.*, n.d.). This large-scale sample collection from salmon migrating through different reaches of the Yukon River system required an experimental validation study to identify the response of heat stress biomarkers in Chinook salmon held at known temperatures for comparison. Traditional laboratory approaches, however, proved problematic given the remoteness of the Yukon River and low probability of adult Chinook salmon survival in transit to a laboratory. The purpose of developing this manipulative thermal challenge protocol was to validate heat stress biomarkers in Yukon River Chinook salmon by holding individuals captive for several hours at a control water temperature that would not induce thermal stress and two elevated water temperatures likely to induce thermal stress. This paper describes the experimental protocol developed in detail, while results of the heat stress biomarkers from the experiment and field collected fish are provided in von Biela *et al*. (n.d.) and an analysis of whole transcriptome for experiment fish is provided in Bowen *et al*. (n.d.).

Here, we tested the hypothesis that a manipulative thermal experiment with large fish could be conducted in a remote field setting with limited equipment. Accordingly, fish were held in a control near ambient river temperature (~14°C) or increased at a fixed rate from river ambient conditions to either low-heat (18°C) or high-heat (21°C) acute treatment. The two temperature treatments were selected for this study based on the available literature for heat stress in Pacific Salmon (Miller *et al.*, 2009; Strange, 2010; Hasler *et al.*, 2012; Hinch *et al.*, 2012; Jeffries *et al.*, 2014) and potential for Yukon River Chinook salmon to encounter the temperature during the seasonal peak of water temperatures in July when much of the population is migrating. The low-heat treatment temperature of 18°C is near the threshold for detecting thermal stress, and the high-heat treatment temperature of 21°C is likely near the upper temperature limit for migrating Chinook salmon (McCullough, 1999). Peak July water temperatures in the Yukon River generally fall between these two treatment temperatures with water temperatures meeting or exceeding 18°C in 85% of years and 21°C in 8% of years at Pilot Station (23 years; 1996–2019, except no data in 2006; data available from the ADF&G AYK Database Management System at https://www.adfg.alaska.gov/CF_R3/external/sites/aykdbms_website). This study was conducted in June, early in the annual summer spawning migration (June–August; McDougall and Lozori 2017), to ensure that individuals had no prior exposure to warm temperature. The need to collect individuals early in the spawning migration from the lower river ruled out the possibility of conducting the experiment at more accessible laboratory facilities further along the migration route. Treatment temperatures in this experiment must be maintained with enough precision and accuracy (±1.0°C) that cellular responses detected in tissue samples by later laboratory analysis could be attributed to specific water temperature levels and differences in cellular biomarkers could be considered interpreted as a thermal stress fingerprint.

## Materials and Methods

This experiment was conducted from 13–21 June 2018, on the bank of the Yukon River at N 61.94716° W 162.84161°, ~1 km upstream of Pilot Station, Alaska, and adjacent to the Alaska Department of Fish and Game (ADF&G) test fishery site. The test fishery is conducted annually to conduct species apportionment in conjunction with sonar counting for managing wild Chinook salmon and Chum salmon (*O. keta*) (Schumann *et al.*, 2017). Pilot Station was identified as the most suitable location for the field experiment because Chinook salmon arrive here early in the season, equipment could arrive by air to Pilot Station, and live fish could be obtained from the ADF&G test fishery for the experiment. The village of Pilot Station is not connected to the road system and barge service along the Yukon River is sporadic, making air cargo the most reliable means of transportation. All equipment was staged and tested in Anchorage, packaged on pallets and shipped to the closest location with large aircraft commercial air cargo service, St. Mary’s, AK (N 62.045305° W 163.218629°). The leg of transportation from St. Mary’s to Pilot Station (N 61.93605°, N 162.88340°) required that all equipment be removed from pallets for a short (<30 min) flight in a small chartered aircraft with a cargo door (Cessna 208 Caravan). Once in Pilot Station, a truck and small aluminum skiff were used to transport equipment from the airstrip to the Yukon River and the Pilot Station sonar and test fishery camp. All components of the experimental system were selected to meet size and weight restrictions of the small charter aircraft.

**Figure 1:**
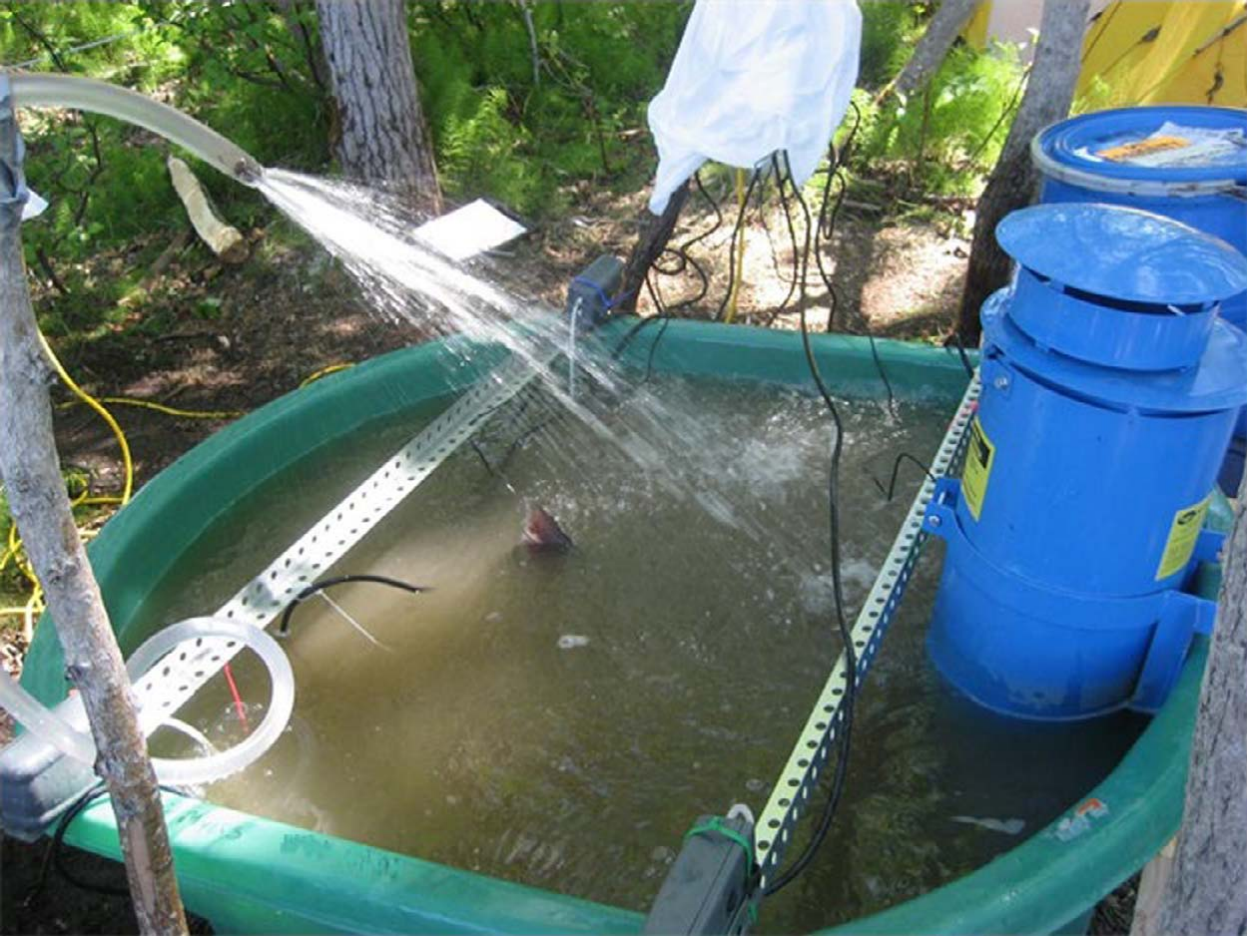
Image of an experimental tank used in the thermal challenge experiment. The upper lobe of a Chinook salmon (*O. tshawytscha*) caudal fin is visible in the center of the water surface. The blue cylinder in the water on the right side of the tank is the primary propane heat source to warm river water to experimental temperature. Additional electric heaters for maintaining consistent experimental temperature are suspended in the water from the perforated crossbars attached to the tank rim. Electronic controls are covered by the white plastic bag in the background as a precaution against moisture from the circulation pump spraying water back into the tank for oxygenation. (Photo courtesy of Shannon Waters, USGS)

Water tanks used in this experiment were 587-L (155 U.S. gallon) oval polyethylene stock tanks commonly used in agriculture (High Country Plastics, model W-155; Fig. 1; $150 USD). A 1.90-cm (0.75-inch) ball valve was plumbed into the drain hole of the tank and connected to a hose running back to the river to allow easier draining. Water was pumped from the river into each tank (*n* = 3) with a gasoline powered pump (Honda WX10T; $460 USD) prior to each experimental trial and drained back into the river by gravity feed at the conclusion of each day.

Initial heating of the experimental tanks from ambient river temperature to experimental temperature was done with a submersible liquid propane (LP) gas stock tank heater (Trojan specialty products model 66B; $475 USD). The LP heater analog thermostat was manually adjusted as necessary to maintain the desired rate of heating. The most consistent results were achieved by increasing the thermostat until the burner of the heater ignited, letting the heater run for 2–3 min, then lowering the thermostat to extinguish the burner and let the warmed water close to the heater circulate through the tank. The target rate of heating for the low-heat (18°C) and high-heat (21°C) treatments was 4°C h^−1^ to minimize total fish holding time out of concern that prolonged confinement may reduce survival. This rate of temperature increase has been used in other heat stress studies in Chinook salmon and justified based on the rapid water temperature changes salmon expose themselves to as they move across thermally heterogeneous habitats near the surface and with depth (Clark *et al.*, 2008 and citations within). Indeed, temperature differences of 4–5°C occur in the Yukon River delta front in June and July, where colder marine and warmer freshwater masses meet (Martin *et al.*, 1986), differences of up to 7°C occur in river networks where tributaries meet the mainstem (Goniea *et al.*, 2006; Keefer *et al.*, 2015), and some adult sockeye salmon in lakes display diurnal vertical migrations that result in 8°C water temperature shifts (Roscoe *et al.*, 2010).

**Figure 2:**
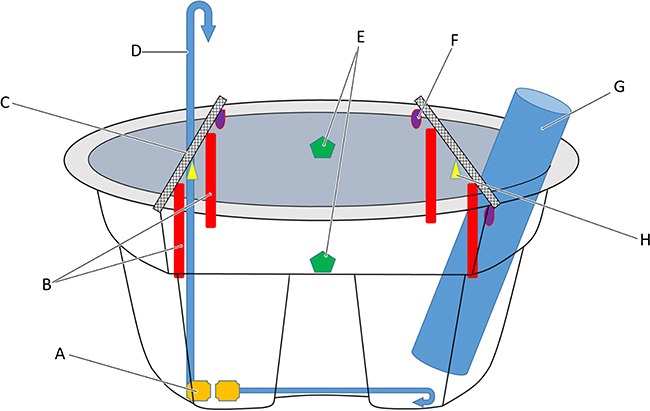
Diagram of how components were positioned in experimental tanks (not to scale). Clockwise from the bottom left: (**A**) Two water pumps (yellow polygon) for both circulating water in the tank and for hose continuously pouring water back into the tank providing aeration. (**B**) Four electric aquarium heaters (red rectangle). (**C**) Two metal crossbars (hatched rectangles) mounted across the tank to support hanging components. (**D**) One vinyl water hose (blue arrow) continually pouring recirculated water back into the tank for aeration. (**E**) Two sensors for digital temperature controllers (green pentagons). (**F**) Three diffusion airstones (purple ovals) from battery powered aerators. (**G**) One submersible propane heater (large blue cylinder) for initial temperature increase. (**H**) Two temperature loggers (yellow triangles). All electric junctions and electronic controls sensitive to water were securely affixed to a tree adjacent to the tank and protected by plastic covering (not shown) and protected by a ground fault circuit interrupter

When the water in each tank approached the desired experimental temperature, the LP heater was shut off and four electric aquarium heaters (Eheim Jager model 3617, 200W; $30 USD) per tank were used to finely control and maintain the desired treatment temperature with two digital heater controllers (Inkbird Tech ITC-306T; $31 USD). These controllers have an LCD display showing current water temperature as well as the programed temperature. The controllers have an integrated switch that activates electrical outlets, turning on the aquarium heaters when the water temperature falls below the programed temperature. Temperature controllers and electric heaters were suspended over the water using metal bars secured across the top of the tanks (Fig. 2). All electricity for the temperature controllers (3 tanks × 2 temperature controllers per tank = 6 temperature controllers) and electric aquarium heaters (3 tanks × 4 heaters per tank = 12 heaters) was supplied by a single gasoline-powered portable generator (Honda EU2200i; $1000 USD), that produced 1800 watts of power.

The experimental design required three Chinook salmon captured each morning by the ADF&G test fishery for 9 days, for a total of 27 individuals. Sample size was limited due to management concerns for Yukon River Chinook salmon. All individuals used in this study were captured using 45.7-m-long by 8.00-m-deep drift gill nets made of double knot multifilament nylon twine with mesh sizes ranging from 6.98 to 21.59 cm (McDougall and Lozori, 2017). Although gillnet capture can be particularly stressful to salmonids, the test fishery has selected multifilament material to reduce stress and mortality. In addition, we intentionally avoided including individuals that were ‘gilled’ with the net wrapped tightly under the operculum and higher chances of injury and stress. Instead, individuals that were less tangled in the mesh and more quickly removed from the net with reduced opportunity for injury and stress were selected. All individuals included in this experiment appeared vigorous.

Following capture, fish were transferred to polyvinyl chloride (PVC) holding tubes in a live well aboard a skiff. The live well was filled with water directly from the river (~14°C). The PVC holding tubes were opaque white, 20 cm in diameter and 90 cm in length, with a series of 2.50-cm holes drilled along the length of the tube and 6.35-mm mesh end caps to allow water to flow through. Keeping the fish in labeled tubes allowed for easy identification and reduced further handling when transferring fish into experimental tanks. Due to the size limitation of the holding tubes and tanks, fish that were >900 mm mid-eye to fork of tail, approximately the 90th percentile in the population, were excluded from the experiment (Jasper and Evenson, 2006). The size of the experimental tanks was limited by the need to fit components into the aircraft (Cessna 208) used to access the experimental location. Different transportation methods may allow for larger scale tanks. Length of the 26 Chinook salmon included in the experiment ranged from 392 to 879 mm (mid-eye to tail fork measurement) with a mean length of 733 mm (SD = 110 mm).

The proximity of the experimental setup location to the ADF&G drift gill net fishing sites minimized the transit time of fish in the live well before introduction into the tanks. The live well was refreshed with fresh river water to maintain oxygen and water temperatures similar to the river whenever the skiff was awaiting an addition fish from the test fishery crew, although oxygen and temperature were not continuously monitored during the short duration when fish were in the live well. Fish spent a mean time of 10 min (SD = 14.5 min) in the live well. However, time varied from a minimum of 2 min to a maximum of 57 min.

Fish were transferred from the live well on the skiff to the experimental tanks immediately upon the skiff’s arrival at the experiment site. Daily assignment of treatments was randomized among tanks, with one fish assigned to the control and each of the two temperature treatments. Since it was rare for all three salmon to be collected in a single drift net set, those captured in close proximity to the experiment site were shuttled back to be placed in the experiment tanks before the skiff returned to await capture of additional fish required for the day. Once all three fish were collected and in the experimental tanks, an additional 30 min was given to recover from capture before heating began. Individual fish usually acclimated quickly, positioning themselves upright in the tanks, typically with their entire body submerged and the tail occasionally breaking the surface of the water. One fish did not recover (i.e. did not maintain equilibrium in the tank) and was removed from the study. Mean total elapsed time fish spent in the experimental tanks was 6:05 hours (SD = 1:01 hours).

River temperature was recorded in the morning and used to determine the temperature of the control tank. Wind across the water surface and the aeration flow tended to cool the water in the control tank so electric heaters were used to maintain stable temperatures. Temperature loggers (Onset TidbiT v2) positioned near and opposite the LP heater recorded temperature every 5 minutes. Prior system testing indicated that water circulation pumps were effective at preventing thermal stratification in the tanks.

Dissolved oxygen concentration was recorded every 30 minutes using a YSI Professional Plus multiparameter meter (YSI Incorporated, Yellow Springs, OH). Dissolved oxygen was supplemented using battery powered aerators and a submersible pump to cycle water at a rate of 30 L min^−1^. The submersible pump outflow was routed through a vinyl hose (1.58 cm inside diameter) to create an ~1-m waterfall back into the tank. Manually agitating the water in the tanks using a bucket was also implemented to supplement dissolved oxygen. Water from the tank was repeatedly scooped and poured out of the bucket from a height of ~1 m for 1 minute of continual agitation every 30 minutes. While this agitation may have resulted in some stress, there was no obvious change in the fishes’ behavior during this process and this concern was outweighed by the need to maintain dissolved oxygen concentrations. Since the agitation was applied equally across treatments such that any potential stress caused by the disturbance would not be a confounding factor for comparison of physiological and cellular responses among groups.

To mitigate against potential toxicity from by-products of fish metabolism (Wicks *et al.*, 2002), a known volume of water in the tank was replaced approximately every 30 minutes with fresh river water. During Days 1, 2 and 3 of the experiment ~ 25% of the water was replaced, but the large influxes of colder river water made maintaining constant experimental temperatures difficult. Eventually the volume of water exchanged was reduced to ~10% of tank volume and frequency of water changes was reduced to once an hour, and only occurred during the initial heating of the tank to experimental temperature. Protocols were consistent across treatments within each experiment day. While we performed water changes as a means of reducing nitrogenous wastes, we did not measure water quality. Future experiments using the methods described above could monitor levels of nitrogenous waste produced as a by-product of fish metabolism to assess if these by-products reach toxic levels.

Visually observing fish while in the tanks was impossible given the turbidity of the Yukon River water (Fig. 1). Unless the fish were seen breaking the water surface, a sweep of the tank by hand every 30 minutes was needed to check if a fish was upright and alive. If a fish was found to have expired or was moribund, the trial was ended for that treatment tank. All fish were sacrificed at the end of the 4-hour treatment period by cranial concussion, cervical dislocation and exsanguination, followed immediately by tissue sample collection.

Temperature data from each trial were assessed to determine success for meeting our desired rate of temperature increase and maintaining target temperatures. The mean temperature of exposure for each individual was calculated as the mean water temperature from both data loggers over the period when water temperature controllers maintained the target temperature (river ambient, 18, or 21°C). The rate of temperature increase (°C h^−1^) was described as the slope from a simple linear regression between temperature (°C) and time (h) for the period from when a fish was placed in the experimental tank until the target temperature was reached and the propane heater was turned off. Rates of temperature increase were compared between the low heat and high heat treatments using a *t*-test to determine if there was a significant difference in heating rate. To assess how well the treatments attained target temperatures, we calculated the mean, standard deviation, minimum and maximum temperature recorded by the temperature loggers, for the period when electric heaters were in use and programed for constant temperatures, until the fish was removed from the tank. In the event of experimental mortalities, *t*-tests were used to compare differences in the length, transit time and the rate of temperature increase between individuals that did and did not survive a treatment.

## Results

**Figure 3:**
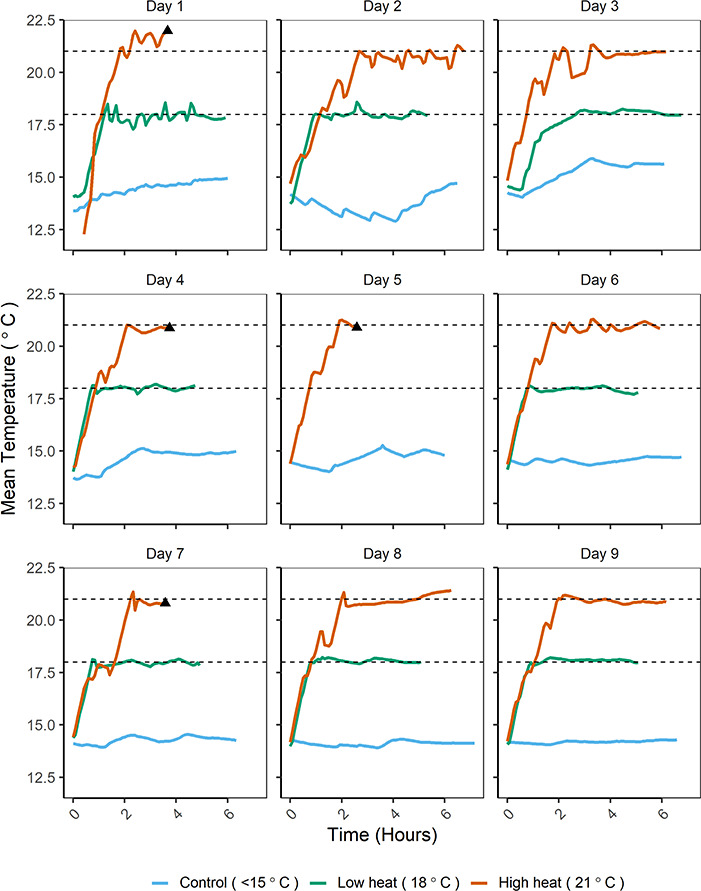
Temperature plots displayed as the mean of two temperature loggers in each experimental tank used to hold adult Chinook salmon. Treatment indicated by line color as control (blue, ambient river temperature), low heat (green) or high heat (orange). Horizontal dashed lines denote the low-heat (18°C) and high-heat (21°C) treatment temperatures. Temperature records for the high-heat treatment are truncated due to fish mortality on Days 1, 4, 5 and 7. The black triangles indicate the points at which fish were found to have expired and the experiment was halted. No temperature records are included for the low heat treatment on Day 5 when the fish did not acclimate and was released.

The rate of temperature increase for low and high treatments was similar among groups (*t*-test, *t* = −1.55, *P* = 0.14) with a mean increase of 3.71°C h ^−1^ (Table 1). Across all days of the experiment, mean holding temperature of the low-heat treatment was 18.0°C (SD = 0.2) and mean holding temperature of the high-heat treatment was 20.9°C (SD = 0.2) (Fig. 3, Table 1). Target temperatures were maintained, with narrow non-overlapping ranges among control (12.9–15.9°C), low-heat (17.3–18.6°C) and high-heat treatments (19.8–22.0°C) (Fig. 3; Table 1).

**Table 1:** Details of timing, duration and water temperature for each fish used in this experiment. Length is the mid-eye to fork to the nearest mm. The time of capture, when an individual was placed in the experimental tank, and the experiment end are reported in local time using 24 h notation. Duration of transport from the time of capture to the time placed in the tank and total time in captivity from capture to sacrifice at experiment completion reported in hours and minutes. Water temperature (°C) mean, min and max columns report mean (± SD), minimum and maximum temperature recorded during the phase of the experiment when propane heating was not in use and the water temperature controllers were used to maintain the target temperature. The increase column reports the rate of temperature increase (°C hr^−1^) when the propane heater was used to raise temperatures to the low-heat (18°C) and high-heat (21°C) target temperatures.

			Local time			Duration		Water temperature				
Treatment	Day	Length	Capture	In tank	End	Transport	Captivity	Increase	Mean ± SD	Min	Max	Note
Control	1	784	11:13	11:40	17:03	0:27	5:50		14.4 ± 0.4	13.4	14.9	
	2	708	9:53	10:00	16:28	0:07	6:35		13.6 ± 0.5	12.9	14.7	
	3	795	11:51	11:57	18:03	0:06	6:12		15.2 ± 0.6	14.0	15.9	
	4	712	10:59	11:03	17:23	0:04	6:24		14.6 ± 0.5	13.7	15.1	
	5	652	11:25	11:27	17:36	0:02	6:11		14.6 ± 0.3	14.0	15.3	
	6	830	9:16	9:21	16:03	0:05	6:47		14.5 ± 0.1	14.3	14.7	
	7	840	11:36	11:38	18:00	0:02	6:24		14.3 ± 0.2	13.9	14.5	
	8	789	9:14	9:18	16:28	0:04	7:14		14.1 ± 0.1	13.9	14.3	
	9	610	9:50	9:52	16:32	0:02	6:42		14.2 ± 0.1	14.1	14.3	
*Mean*		*747*	*10:35*	*10:41*	*17:04*	*0:06*	*6:28*		*14.4* ± 0.4	*12.9*	*15.9*	
Low heat (18°C)	1	806	10:56	11:52	18:17	0:56	7:21	3.38	17.9 ± 0.3	17.3	18.6	
	2	774	10:54	11:01	16:56	0:07	6:02	4.66	18.0 ± 0.2	17.7	18.6	
	3	740	11:26	11:43	18:30	0:17	7:04	1.63	18.1 ± 0.1	18.0	18.2	
	4	850	11:40	11:46	17:02	0:06	5:22	5.75	18.0 ± 0.1	17.7	18.2	
	5	N/A	11:43	11:46	N/A	0:03	Fish released					
	6	790	9:59	10:02	15:34	0:03	5:35	5.29	17.9 ± 0.1	17.7	20.7	
	7	690	11:36	11:39	17:09	0:03	5:33	5.10	17.9 ± 0.1	17.7	20.5	
	8	799	9:49	9:52	15:41	0:03	5:52	4.84	18.1 ± 0.1	17.9	20.7	
	9	590	10:05	10:07	16:18	0:02	6:13	3.19	18.1 ± 0.0	18.0	20.7	
*Mean*		*754*	*10:54*	*11:05*	*16:55*	*0:11*	*6:07*	*4.23*	*18.0* ± 0.2	*17.3*	*18.6*	
High heat (21°C)	1	740	10:55	11:52	15:47	0:57	4:52	6.24	21.5 ± 0.4	20.7	22.0	Mortality
	2	619	9:16	9:26	16:44	0:10	7:28	2.12	20.7 ± 0.2	20.2	21.3	
	3	833	11:02	11:17	17:39	0:15	6:37	2.78	20.8 ± 0.4	19.8	21.3	
	4	392	10:10	10:17	14:41	0:07	4:31	3.00	20.8 ± 0.1	20.6	21.0	Mortality
	5	605	10:57	11:00	14:02	0:03	3:05	3.27	21.1 ± 0.1	20.9	21.2	Mortality
	6	713	9:15	9:21	15:45	0:06	6:30	3.67	20.7 ± 0.2	20.7	21.3	
	7	879	11:10	11:16	15:19	0:06	4:09	2.14	20.5 ± 0.2	20.5	21.3	Mortality
	8	858	9:13	9:18	16:06	0:05	6:53	2.79	20.7 ± 0.2	20.7	21.4	
	9	650	10:49	10:51	17:41	0:02	6:52	3.18	20.7 ± 0.1	20.7	21.2	
*Mean*		*699*	*10:18*	*10:30*	*15:58*	*0:12*	*5:39*	*3.24*	*21.0 ± 0.2*	*19.8*	*22.0*	

Dissolved oxygen concentrations for all three treatments spanned the following means and range: control mean, 8.97 mg L^−1^ (range, 7.03–10.5 mg L^−1^); low-heat mean, 8.33 mg L^−1^ (range, 6.52–10.3 mg L^−1^); and high-heat mean, 8.14 mg L^−1^ (range, 6.69–10.5 mg L^−1^). Mean ambient dissolved oxygen concentration measured in the river adjacent to the experiment site was 9.33 mg L^−1^ (range, 8.29–9.87 mg L^−1^). There was not a statistically significant difference among DO concentration in the control group and the two treatment groups (ANOVA, *P* = 0.42, *F* = 0.86). All water temperature and dissolved oxygen data and metadata are available in von Biela and Donnelly (2020).

All fish in the low-heat and control treatments survived to the end of the trial for survival rates of 100% (eight in the low-heat treatment and nine in the control). There were four mortalities in the high-heat treatment (Days 1, 4, 5, 7). Death occurred after reaching experimental temperature, but prior to the end of the 4-hour trial period for a survival rate of 56% (*n* = 9). Three fish were found dead on the bottom of the tank during periodic checks and were sampled immediately. A fourth fish on Day 7 was found unresponsive with virtually no gill movement, at which time the trial was ended. Survival in the high heat treatment was not related to body length (*P* = 0.47, *t* = 0.76), transit time (*P* = 0.39, *t =* 0.91) or the rate of temperature increase (*P* = 0.40, *t* = 0.90).

## Discussion

The portable experimental system described in this protocol successfully manipulated and maintained water temperatures and allowed for an experimental study on large-bodied fish in a remote setting. Across all treatments, the protocol developed was both more accurate and precise for holding temperature than increasing temperatures. Temperatures could be maintained with digital temperature controllers that automatically turned electric heaters on and off within 0.1°C of a target temperature. An improvement in holding temperature precision was indicated by reduced standard deviations after the first 3 days that was related to discontinuing water changes in the temperature holding phase of the experiment. In comparison, increasing water temperatures from ambient required the use of an additional propane-fueled heating source with a less precise analog thermostat and resulted in wider variation in the rate of warming among trials. We are unaware of any product that would have allowed improvement in our ability to raise temperatures in a more uniform, consistent pattern given the limitations of our field setting. Further, water changes during the warming period impeded uniform temperature increases.

The capture, transport and confinement of Chinook salmon during this experiment are likely still stressful despite any mitigation measures (Portz *et al.*, 2006) and result in inherent limitations of this and any other experimental protocols. First, the physiological parameters measured in the control group do not reflect unstressed fish, but rather provide an opportunity for comparison to treatment groups with increased water temperature. Second, the contrast in physiological parameters between the control and an elevated water temperature treatment group reflects the difference in water temperature only if it is reasonable to conclude there is no other consistent difference between the groups exist that confound the comparison. Third, it is important to recognize that experimental protocols and the resulting experimental data inevitably resulted in a multiple stressors scenario with the potentially interactive effects among intended (e.g. water temperature) and unintended (e.g. capture and handling) stressors (Meka and McCormick, 2005; Cooke *et al.*, 2013). These interactions may complicate the comparison of physiological parameters among experimental and field-sampled fish. Because multiple stressors typically exacerbate any physiological responses through synergistic effects (Cooke *et al.*, 2013; Gale *et al.*, 2013), experimentally derived indicators of stress risk underestimating stress in field-sampled fish.

Future field experiments could consider additional methods to ameliorate the effects of captive handling stress. For example, in this protocol we opted for a relatively rapid increase in water temperature and short recovery time to minimize the length of confinement given the uncertainty of adult Chinook salmon surviving in a relatively small tank. Given that all individuals in the control and low heat groups survived, a slower temperature increase and longer recovery period in the experimental tank prior to increasing the water temperature appears to be possible and may reduce the interactive multiple stressor effect by allowing Pacific salmon to more fully recover from capture and handling stress. The addition of a mild saline solution to holding tank water is commonly used to lower osmoregulatory imbalance associated with handling stress in several fish species (Schreck, 1982; Barton and Zitzow, 1995; Swanson *et al.*, 1996) and may prove helpful as well.

Mortality only occurred during the high-heat treatment, which likely reflected the combined effects of thermal and captive handling stress. Indeed, McCullough’s (1999) review suggested that the high-heat temperature of 21°C, represents the upper temperature limit for migrating Chinook salmon across all populations. Continued research has revealed adaptive variation in the upper thermal limits among populations (Eliason *et al.*, 2011) and a study in the Klamath River, OR, USA, provides example of a population that has an upper thermal limit closer to 23°C (Strange, 2010). In the absence of a study that identified the upper temperature limit of Yukon River Chinook salmon, it is reasonable to conclude that McCullough’s (1999) 21°C limit is applicable given that Yukon River water temperatures are predominantly below 21°C and offer little opportunity for adaptation. The samples from this experiment did ultimately provide evidence of heat stress in the 21°C treatment (Bowen *et al.*, n.d.; von Biela *et al.*, n.d.), and high rate of experimental mortality reported here. In all cases of mortalities, fish sank to the bottom of the tank and showed no visible signs of distress (i.e. splashing, gulping at the surface). These observations are consistent with those reported for Fraser River sockeye salmon even in years when estimates of premature mortality were high (Hinch and Martins, 2011). The high level of experimental mortality is similar to observations from Pacific salmon in natural systems at the same temperatures (Keefer *et al.*, 2010; Hinch and Martins, 2011; Hinch *et al.*, 2012). Mortalities occurred across a range of days and spanned the variation in transport times and rates of increasing temperature (Table 1). Although this experiment was not designed to examine survival outcomes and these results carry the caveat of small sample size (*n* = 9), it is worth noting that survival was not clearly tied to differences in body length, transit time or the rate of temperature increase.

Two unexpected challenges were encountered during the development of this protocol. First, adult salmonids are often thought to be calmer when an enclosure prevents movement, as demonstrated by the widespread use of holding tubes and cradles to restrain fish without chemical sedation and with negligible mortality (Larson, 1995; van Poorten and Post, 2005; Spromberg *et al.*, 2016). Yet, Chinook salmon used in a 1-day pilot trial prior to the beginning of this experiment had difficulty remaining upright while in the 20-cm diameter tubes, and all three individuals confined to PVC holding tubes for the duration of the trial died or were moribund at the end of the day. After refining the protocol to use PVC tubes only for transportation, all fish in the control and low-heat groups survived to the end of the trial period. We do not recommend confining adult Chinook salmon in holding tubes for prolonged periods.

Second, salmon used in our trial consumed dissolved oxygen at a faster rate than expected despite low activity levels. It was necessary to add more aerators than planned and supplement with manual bucket agitation to maintain levels of dissolved oxygen. While the levels of dissolved oxygen were generally above the Alaska Department of Environmental Conservation guideline of 7 mg L^−1^, the dissolved oxygen levels were still likely low enough to contribute to stress. Oxygen requirements increase with water temperature and vary among species and population within of Pacific salmon based on local adaptations to conditions normally encountered on upstream migrations (Clark *et al.*, 2008; Eliason *et al.*, 2011). Thus, future refinement of this protocol should attempt to provide a higher concentration of dissolved oxygen through several large aerators and high-quality air stones or diffusion of pure oxygen into water (if transportation of pressurized gas cylinders is available). While not planned, the lower levels of declined oxygen probably mimic a similar *in situ* decline in dissolved oxygen with warming water temperature in rivers.

This relatively simple protocol is designed to encourage more researchers to consider the possibility of conducting remote field experiments with adult salmonids across their northern range extent even when locations are remote and typical laboratory conditions cannot be strictly achieved. The component pieces can be transported via boat, aircraft or truck, have a small footprint, are quickly assembled and do not require access to utilities. Target temperatures and the rate of temperature increase are easily modified to ecologically relevant values that address questions specific to a study species and ecosystems. Slight modifications to the temperature controller (e.g. Inkbird Tech ITC-308) could also accommodate chillers to reduce or maintain temperatures as needed for specific questions or to accommodate work in more temperate climates with warmer air temperatures.

## Funding

This work was supported by the U.S. Geological Survey Ecosystems Mission Area and the Arctic-Yukon-Kuskokwim Sustainable Salmon Initiative (grant number 1611).
